# Postoperative Pulmonary Dysfunction and Mechanical Ventilation in Cardiac Surgery

**DOI:** 10.1155/2015/420513

**Published:** 2015-02-03

**Authors:** Rafael Badenes, Angels Lozano, F. Javier Belda

**Affiliations:** Department of Anesthesiology and Surgical Intensive Care, Hospital Clinic Universitari de Valencia, University of Valencia, 46010 Valencia, Spain

## Abstract

Postoperative pulmonary dysfunction (PPD) is a frequent and significant complication after cardiac surgery. It contributes to morbidity and mortality and increases hospitalization stay and its associated costs. Its pathogenesis is not clear but it seems to be related to the development of a systemic inflammatory response with a subsequent pulmonary inflammation. Many factors have been described to contribute to this inflammatory response, including surgical procedure with sternotomy incision, effects of general anesthesia, topical cooling, and extracorporeal circulation (ECC) and mechanical ventilation (VM). Protective ventilation strategies can reduce the incidence of atelectasis (which still remains one of the principal causes of PDD) and pulmonary infections in surgical patients. In this way, the open lung approach (OLA), a protective ventilation strategy, has demonstrated attenuating the inflammatory response and improving gas exchange parameters and postoperative pulmonary functions with a better residual functional capacity (FRC) when compared with a conventional ventilatory strategy. Additionally, maintaining low frequency ventilation during ECC was shown to decrease the incidence of PDD after cardiac surgery, preserving lung function.

## 1. Introduction

Postoperative pulmonary dysfunction (PPD) is a quite common complication after cardiac surgery [1]; 40% of patients readmitted into intensive care units (ICU) present respiratory failure [2], and the adequate therapeutic management that might reduce its incidence is still unknown.

PPD pathophysiology is complex and its mechanisms are not clear (Table 1). Even so, there are many surgery-related factors that predispose cardiac surgical patients to the pathogenesis of postoperative pulmonary complications, such as the effects of general anaesthesia combined with the effects of median sternotomy incision, cardiopulmonary bypass (CBP), internal mammary artery dissection, and the use of topical cooling for myocardial protection [3].

PPD clinical manifestations include pleural effusion, with a frequent presentation (27–95%) [4], and atelectasis (16.6–88%) [5] and postoperative hypoxemia without clinical symptoms (3–10%) [6] and acute respiratory distress syndrome (ARDS), which have a low incidence (0.5–1.7%) [7] but high mortality (50–90%) [8].

Furthermore, cardiac surgery produces a whole body inflammatory response that has been highly related with lung injury [9]. This systemic inflammatory response is associated with anomalies in gas exchange, such as an increased pulmonary shunt fraction [10], increased pulmonary vascular resistance [11], and intrapulmonary aggregation of leukocytes and platelets [12]; also with alterations in lung mechanics, resulting in a reduced pulmonary compliance and reduced functional residual capacity (FRC) and vital capacity (VC) or both of them.

## 2. Factors Associated with the Development of PPD after Cardiac Surgery

### 2.1. General Anesthesia

Is well known that lung functional impairment is inevitable after any major surgery, a condition that most likely is related to the general anesthesia.

There are many factors related with general anesthesia that affect pulmonary function. Anesthesia with the prolonged supine position produces an upward shift of the diaphragm; this, combined with the relaxation of the chest wall and altered chest wall compliance, and with the blood volume modification in the thorax, results in an alteration of the ventilation-perfusion mismatch and an abnormal pulmonary shunt fraction [13]. Furthermore, the majority of the drugs used in anesthesia also have repercussion in the pulmonary function; inhalation anesthetics, for example, inhibit hypoxic pulmonary vasoconstriction, and narcotics reduce hypoxic and hypercapnic ventilatory response.

All these combined factors result in a widened alveolar-arterial oxygen gradient [14] and a reduction of the vital capacity and the functional residual capacity of the lungs [13]; and it also contributes to the onset of hypoxemia and atelectasis [14].

### 2.2. Extracorporeal Circulation (ECC)

Extracorporeal circulation (on-pump surgery) has clear consequences for postoperative pulmonary function. The vascular contribution to the lungs depends almost exclusively on the pulmonary arteries. The principal function of the bronchial circulation is to feed the pulmonary structures; thus, it is responsible for approximately 1% of the pulmonary circulation. However, when the arterial circulation is chronically compromised, the bronchial circulation takes on a leading role.

When ECC initiates, the cessation of pulmonary ventilation results in collapsed lungs with loss of surfactant and alveolar collapse, favouring the retention of secretions and atelectasis. Moreover, pulmonary circulation is stopped resulting in a pulmonary ischemia with injured capillary walls and the release of inflammatory mediators [15]. All of this increases abnormalities in gas exchange and leads to closure of the small airways.

Thus, compared to on-pump, off-pump surgery was associated with a reduced inflammatory response and lower levels of circulating neutrophils and monocytes [16].

Furthermore, many studies have showed that procedures without EC have lower pulmonary complication rates, earlier extubations, shorter MV duration, and a lower incidence of pneumonia compared to those with ECC [17].

So it seems to be clear that the use of ECC has evident consequences for postoperative pulmonary function compared with other types of major surgery, and it appears to cause additional lung injury and a delay in pulmonary recovery, probably due to the damaging effects of the associated systemic inflammatory response.

On the other hand, some studies have found that off-pump surgery was not always more beneficial than on-pump surgery, showing, as Groeneveld et al. demonstrated, that ECC is not always a determinant for the development of PDD [18].

### 2.3. Surgical Effects and Systemic Temperature

There are some factors associated exclusively with cardiac surgery that affects pulmonary function and contribute to de development of PPD, like the median sternotomy incision, hypothermia for myocardial protection, dissection of the internal mammary artery, and the use of cardiopulmonary bypass.

It is not clear the effect of median sternotomy incision on PPD. Studies comparing sternotomy incision with thoracotomy incision showed that the minimal interruption to the chest wall, less trauma, and negligible lung compression make sternotomy a relatively benign procedure [14]. Many other studies, like the ones from Barnas et al. [19] or Ranieri et al. [20], have also demonstrated that the sternotomy incision did not affect the mechanical properties of the chest wall.

Although patients with normothermia exhibited decreases in the shunt fraction, PA-a O_2_, and the alveolus-arterial gradient of CO_2_; central temperature did not appear to significantly influence gas exchange (alveolar arterial difference in oxygen partial pressure, or PA-a O_2_) after an aortocoronary bypass graft. It suggests that normothermia might be beneficial in the preservation of pulmonary function after cardiopulmonary bypass surgery [21].

With all this, it seems clear that the severe pulmonary dysfunction developed after cardiac surgery is influenced by two main factors. One is the mechanical stress and biotrauma induced by the mechanical ventilation and the use of an inadequate ventilatory strategy with high volumes and low PEEP levels that stimulate atelectasis. Second is the exaggerated systemic inflammatory response to the cardiac surgery and its associated factors, like the effects of general anesthesia, sternotomy incision, topical cooling, and extracorporeal circulation.

### 2.4. Mechanical Ventilation (MV)

Mechanical ventilation can cause significant changes in lung structure and function. This lung injury during mechanical ventilation induces pulmonary inflammation that can spread to distant organs and considerably affect treatment outcomes [22].

In addition to the MV lung injury, there are many factors involved in cardiac surgery that affect pulmonary function too and play an important role in this inflammatory response, including extra corporeal circulation (ECC), the surgical intervention, and injuries due to ischemia-reperfusion.

Ventilation-induced lung injury with pulmonary inflammation results from both mechanical and biological trauma [23].

Mechanical trauma involves both volutrauma and barotrauma. The term barotrauma is used to indicate lung damage attributable to the application of high airway pressure [24]; in this way, volutrauma is referred to the alveolar overdistension when using large tidal volumes. The stress produced by this mechanical trauma could be strong enough to cause destruction of the anatomical lung structure with epithelial injury, loss of epithelial integrity, and edema.

Biological trauma is referred to the biological reaction in response to mechanical ventilation stress. Ventilating with high tidal volume induces the release of inflammatory mediators that contribute to this ventilation-induced biotrauma [25] by activating both local and systemic inflammatory responses, what causes the release of cytokines and other soluble inflammatory mediators and the activation of complement, leukocytes, and endothelial cells, resulting in an alteration of the normal function of tissues and organs by altering cellular pathways.

Biotrauma results from the forces acting during mechanic ventilation with the cyclic opening and collapse of alveoli and its overdistension that induce the release of proinflammatory cytokines, recruitment of leucocytes, and local initiation of inflammatory processes. It still remains unclear how mechanical forces are translated into the biochemical signals that produce biotrauma [26]. According to the experimental studies, that have studied the relationship between mechanical distension of the alveolocapillary membrane and the production of mediators, the biotrauma hypothesis assumes that lung injury is caused by the release of this proinflammatory mediators and the excessive activation of the immune system; in this way, theories proposed imply mechanoreceptors, stretch-sensitive channels, activation of inflammatory cascade [27], and activation of the transcription of the nuclear factor kappa [28] (NF-*κ*B) which becomes the major factor of modification of nucleic acid sequence in the cell nucleus and synthesis of inflammatory factors (TNF-*α*, IL-1*β*, IL-6, and IL-8) [26].

Biotrauma causes diverse biological responses, like the action of oxygen free radicals, cellular mechanisms of growth or division and apoptosis, altered expression of genes and proteins, activation of coagulation cascade, and stimulation of various elements of the immune system, which lead consequently to the cascade of inflammation. And this exaggerated inflammatory response initiated locally in the pulmonary tissue may cross into the systemic circulation causing systemic inflammatory response [29].

The presence of atelectasis is one of the principal causes of PDD and a primary factor in the development of pulmonary inflammation [27], and there is a correlation between the amount of atelectasis and the intrapulmonary shunt [30].

Atelectasis is related with a too low end-expiratory lung volume and their development is associated with the loss of surfactant and the cyclic opening and collapse of unstable lung units, which is promoted by ventilation with zero or inadequate PEEP [31]. This repetitive collapse and reopening of alveoli is termed atelectrauma [32].

When the atelectatic lung units are exposed to high ventilating pressures, the alternate opening and collapsing of alveoli generate damaging transverse forces localised in these dependent parts. This transverse forces applied to the collapsed units could be sufficiently high to damage the airway epithelium and cause a “stress induced failure” of the alveolar capillary membrane resulting in an increased microvascular permeability, edema, and an influx of plasmatic proteins causing surfactant dysfunction and initiating an inflammatory reaction [33–35]. In this way, Dreyfuss et al. [35] demonstrated that volutrauma (ventilation with high tidal volumes producing high transpulmonary pressure) rather than barotrauma (ventilation with high pressures producing low transpulmonar pressure) was the primary determinant factor for pulmonary injury and inflammatory response.

## 3. Therapeutic Measures That Minimize PPD after Cardiac Surgery

### 3.1. Protective Ventilation Strategy: Open Lung Approach (OLA)

It is well studied that protective ventilation strategies can reduce the incidence of atelectasis and pulmonary infections in surgical patients [36]. The OLA ventilatory strategy was initially conceived to treat patients with ARS; its aim is to reduce the shear forces generated by the cyclic opening and closing of the atelectasic alveoli and minimize the development of diffuse alveolar damage, pulmonary edema, recruitment of inflammatory cells, and cytokines production [37, 38] of the injurious ventilation strategies with high tidal volume.

The OLA strategy has to be applied with recruiting maneuvers and sufficient PEEP to increase transpulmonary pressure enough for maintaining opened the maximum possible number of alveoli with minimum delta pressure (Pplateau-PEEP) to prevent pulmonary overdistension [39]. The low delta pressure is typically achieved by using low tidal volumes (4–6 mL/kg). Serita et al. [40] found that individualised recruitment maneuvers, brought about an improvement in oxygenation and lung compliance in patients undergoing selective cardiac surgery.

Using OLA ventilatory strategy the sudden changes of volume in large alveolar zones are minimized [41] and atelectasis were not observed in CT-scans in healthy anesthetized children. This ventilatory strategy also attenuates the surfactant alterations, what in consequence reduce the loss of proteins in the alveoli [42].

As this strategy prevents the cyclic collapse of alveoli by splinting them open at end-expiration, the stress to the alveolocapillary membrane resulted to be limited. It was observed a decrease in the biochemical markers released by damaged cells after ventilation with high pressures that confirm this [43].

It is important to notice that applying an adequate PEEP level and preventing the collapse at end-expiration minimizes the inflammatory response and diminishes bacterial translocation [44]. Furthermore, two recent studies with patients undergoing abdominal surgery showed that pulmonary inflammation can be reduced [45] and procoagulant alveolar changes can be prevented [46] by using lower tidal volume and PEEP.

As many studies have demonstrated, OLA strategy has multiple advantages. Miranda et al. [47] showed that OLA ventilation (tidal volume 6 mL/kg, PEEP 14 cm H_2_O), applied immediately after intubation, significantly attenuates the inflammatory response by reducing IL-6, IL-8, IL-10, TNF-alpha, and interferon-gamma plasma levels compared to conventional ventilation (tidal volume 8 mL/kg, PEEP 5 cm H_2_O). During mechanical ventilation, the application of OLA was accompanied by significant increases in the PaO_2_/FiO_2_, suggesting a significant reduction in atelectasis [48]. These investigators found later [49] that the effect of OLA on pulmonary volume was maintained after extubation; the day after extubation, the group ventilated with OLA showed 40% higher FRC than those given conventional ventilation. This effect on the FRC was maintained until the 5th day after extubation. Also, the OLA group had a significant decrease in the incidence of hypoxemia (SpO_2_ < 90% with ambient air) on the day after extubation compared to the group with conventional ventilation.

Furthermore, Ranieri et al. [50] found in previous experimental findings in patients with ARDS, that the levels of tumor necrosis factor-alpha (TNF*α*), interleukin-6 (IL-6), and IL-8 in bronchoalveolar lavage (BAL) were lower with a ventilatory strategy titrated for optimal positive end-expiratory pressure (PEEP) and low tidal volumes than with a strategy that used high tidal volumes. In a multicentre study with 861 patients, ventilation with low tidal volumes (6 mL/kg) diminished plasma concentrations of IL-6 and significantly reduced the 28-day mortality of patients with ARDS. This suggested that the application of suitable ventilatory strategies clearly affected the development of an inflammatory response after cardiac surgery.

The OLA strategy has not been evaluated clinically in terms of outcomes (mortality or readmissions to the ICU). Even though, when the causes for readmission into the ICU after cardiac surgery were studied, Chung et al. [51] founded that after discharge from the ICU, the percent increase in the required fraction of inspired oxygen was correlated to an increased risk of readmission. Given that OLA strategy reduces the incidence of hypoxemia and increases FRC on discharge, these results suggested that it might reduce the incidence of ICU readmission.

On the other hand, OLA ventilatory strategy has some adverse effects that have been noticed. For example, high PEEP may detrimentally increase intracranial pressure and impair ventricular filling, the RV afterload is increased, but contractility is not affected [52, 53].

Cardiovascular effects are particularly prominent in patients who are fluid depleted.

Dyhr et al. [54] found that cardiac output was not affected by high PEEP levels after a recruitment maneuver in cardiac surgery patients. These results were later confirmed by Miranda et al. [55] who, using a pulmonary artery catheter in patients undergoing cardiac surgery, showed that OLA ventilation did not affect pulmonary vascular resistance or the RV ejection fraction.

As we said, high PEEP could increase RV afterload. Even so, high PEEP levels used during OLA ventilation probably did not affect RV afterload because atelectasis was avoided and low tidal volumes were used. This is because it has been shown that atelectasis caused a significant increase in RV afterload [56]. This effect is because of two different mechanisms, the local hypoxic pulmonary vasoconstriction induced in nonaerated lung areas [53] and the capillary compression due to the overdistension in aerated lung areas.

Several clinical and experimental studies suggested that OLA strategy and isolated recruitment maneuvers increased RV afterload in patients without a history of RV failure undergoing cardiac surgery [57, 58]. Additionally, Miranda et al. [55] showed, at the study we have mentioned before, that high PEEP levels during OLA ventilation did not decrease the RV preload when the patients had an adequate previous preload.

Even so, an exhaustive monitoring is absolutely necessary when isolated recruitment maneuvers are performed in patients with previous right cardiac failure. It is important to be extremely cautious with these patients, and avoid them if adverse events are predictable according to the monitored values.

Taking all these into account, our group recommends OLA strategy, initiating it after the intubation in the OR and continuing with this strategy until the patient is extubated. We support OLA in this context, as we have not found adverse effects and, as we have mentioned before, it has important potential advantages (like reducing ventilator-induced pulmonary inflammation, increasing the PaO_2_/FiO_2_, attenuating the postoperative reduction in FRC, and decreasing the incidence of hypoxemia).

### 3.2. Ventilation Strategy during ECC

Apnoea during ECC has been suggested to promote activation of lysosomal enzymes in the pulmonary circulation, which in turn are correlated with the incidence of postoperative pulmonary dysfunction [59]. Hipoventilation during ECC is associated with the development of microatelectasis, hydrostatic pulmonary edema, poor compliance, and an increased incidence of infection [60]. In this way, the hypothesis is that maintaining mechanical ventilation during ECC may limit postoperative pulmonary complications [61].

Atelectasis is the principal determinant in postoperative lung gas exchange and may play a larger role in ventilatory abnormalities after cardiac surgery than edema due to increased permeability.

To prevent all these complications, it has been applied some maneuvers such as the intermittent ventilation or application of continuous airway pressure (CPAP) during ECC [62, 63]. CPAP application during CPB has been reported as an effective adjunct in some studies [63]. Loeckinger et al. [61] studied continuous positive airway pressure (CPAP) at 10 cm H_2_O during ECC and the effect on postoperative pulmonary gas exchange. They found a significantly higher PaO_2_, a significantly lower PA-a O_2_ 4 h after ECC, and better gas exchange after extubation in the CPAP group compared to controls. More recently, John and Ervine [64] demonstrated in their randomized study that maintaining ventilation with a tidal volume of 5 mL/kg during ECC provided other benefits compared to discontinued ventilation. They showed a decrease in extravascular lung water and a shorter extubation time in the ventilation group compared to controls. In this way, Davoudi et al. [65] showed in a prospective randomized study that a continued ventilation with low tidal volume during CPB improved post-by pass oxygenation and lung mechanics.

On the other hand, even though the use of CPAP [61], recruitment maneuvers [66], or low-tidal volume ventilation during CBP has demonstrated to decrease inflammation and improve oxygenation, lung mechanics, and shunt fraction, this positive effects have been shown to be transient with a questionable impact on the clinical outcome [67].

Another option proposed to attenuate lung dysfunction post-ECC is maintaining ventilation together with pulmonary artery perfusion during ECC. In this way, Friedman et al. [68] demonstrated in an experimental comparative study that ventilation with pulmonary artery perfusion during ECC might have benefits in preserving lung function by reducing platelet and neutrophil sequestration and attenuating the TXB2 response after ECC. On the other hand, another experimental study by Serraf et al. [69] showed no significant improvement in pulmonary vascular resistance, respiratory index, or oxygen tensions with continuous ventilation during ECC.

Based on these results, the evidence for clear benefits of maintining ventilation during ECC is not totally clear. Even so, continued ventilation with low frequency during ECC seems to be easy, safe, low-cost, and potentially quite beneficial and it has been suggested to be an easy method to implement with no additional cost.

### 3.3. Early Extubation and Noninvasive Mechanical Ventilation

As we said before, ventilation with a high tidal volume and low or none PEEP, in cardiac surgery patients, is associated with an inflammatory response that contributes to the ventilation induced biotrauma [25]. High tidal volume ventilation in the immediate postoperative period of cardiac surgery has been associated with prolonged mechanical ventilation, a longer ICU stay and an increased risk of organ dysfunction [70]. In this way, using low tidal volumes (of around 6 mL/Kg) and an adequate PEEP level in the intraoperative and postoperative period have been highly recommended to avoid lung collapse and diminished atelectasis. This could also reduce duration of mechanical ventilation and the reintubation rate [71].

Early extubation (less than 6–8 h) after cardiac surgery has been shown to reduce complications in the postoperative period, as well as decrease in ICU stay and costs [69].

Camp et al. [72] demonstrated that an early extubation (within 9 hours after cardiac surgery) is associated with an improved outcome and have been shown to be the best predictor of uncomplicated recovery and a decreased late mortality after cardiac surgery.

Furthermore, a recent Cochrane review [73] concluded that early intubation is not associated with an increased risk of postoperative complications or reintubation and it produces a reduction of the length of ICU stay.

Atelectasis plays an important role in the development of postoperative respiratory failure [74]. Noninvasive mechanical ventilation (NIV) should be applied to prevent acute respiratory failure (ARF) in patients at high risk of developing it [75].

NIV has also been used to treat an established postoperative acute respiratory failure, although there are non-conclusive results [76] and it has not demonstrated to be clearly effective once ARF is already established [77].

Otherwise, preventive use of NIV or CPAP has demonstrated to reduce respiratory work and improve gas exchange, oxygenation and alveolar ventilation [78–80], and it could also be used to wean patients from mechanical ventilation [75]. If it is used correctly, NIV has been reported to reduce atelectasis and PPD, and to diminish reintubation rates, length of stay in ICU, and hospital and ICU readmissions [79, 80].

## 4. Conclusion

PPD is a frequent and almost inevitable consequence of cardiac surgery whose incidence still remains unacceptably high nowadays. His pathogenesis is not clear, but many factors have been shown to be involved in its development. In this way, there are two principal mechanisms that have been identified as the fundamental causes for the development of PPD, one is the stress of the surgery and its associated factors (ECC, median sternotomy incision, hypothermia for myocardial protection, dissection of the internal mammary artery, etc.) that cause an important systemic inflammatory response. The other important factor is the lung injury caused by inflammation and aggravated by suboptimal mechanical ventilation.

Taking all this in to account, our group recommends the use of OLA strategy in patients undergoing cardiac surgery with an early initiation (after the orotracheal intubation), combining low tidal volumes (tidal volume 6 mL/kg) with recruitment maneuvers and the instauration of a PEEP of 8–14 cm H_2_O. Additionally, maintaining low frequency ventilation during ECC seems to be a quite promising strategy with important benefits in preserving lung function. With these two ventilatory procedures, we could probably attenuated the inflammatory response, improve gas exchange parameters and postoperative pulmonary functions with a better FRC, and reduce the incidence of readmission in UCI with a better outcome. And all these benefits could be achieved with almost a little or even no hemodynamic alterations.

Early extubation is well documented and should be the goal in adults after cardiac surgery, as it may reduce postoperative complications and decrease ICU stay and costs.

NIV applied early has shown to be effective in reducing atelectasias and PPD, minimising reintubation rates, length of stay in ICU, and hospital and ICU readmissions.

## Figures and Tables

**Table 1:** Pathogenetic mechanisms of postoperative pulmonary dysfunction (PPD).

Specific to cardiac surgery:	
(i) Median sternotomy incision	
(ii) Use of cardiopulmonary bypass (CPB)	
(iii) Transfusion of blood product	
(iv) Topical cooling for myocardial protection	
(v) Dissection of the internal mammary artery	
(vi) Effects of general anesthesia	

Anomalies in gas exchange:	
(i) Widening of the alveolar-arterial oxygen gradient	
(ii) Increased microvascular permeability in the lung	
(iii) Increased pulmonary vascular resistance	
(iv) Increased pulmonary shunt fraction	
(v) Intrapulmonary aggregation of leukocytes and platelets	

Alterations in lung mechanics:	
(i) Reductions in vital capacity (VC)	
(ii) Reduction of functional residual capacity (FRC)	
(iii) Reduction of static and dynamic lung compliance	
